# Hemorrhagic cystitis induced by intravenous esketamine in the treatment of new-onset refractory status epilepticus in children: a case report

**DOI:** 10.3389/fped.2025.1662542

**Published:** 2025-10-03

**Authors:** Jin-Meng Ding, Hong-Jun Miao, Yao Liu

**Affiliations:** ^1^ Department of Pharmacy, The Affiliated Taizhou People's Hospital of Nanjing Medical University, Taizhou, China; ^2^ Department of Emergency, Children's Hospital of Nanjing Medical University, Nanjing, China; ^3^ Department of Pharmacy, Children's Hospital of Nanjing Medical University, Nanjing, China

**Keywords:** esketamine, new-onset refractory status epilepticus, pediatric, ketamine-associated cystitis, hematuria

## Abstract

**Introduction:**

Esketamine, the S-enantiomer of ketamine, has been considered for terminating new-onset refractory status epilepticus (NORSE). However, there is limited large-scale data on its safety and comparative effectiveness. Ketamine-associated cystitis (KAC) is a known complication of chronic recreational ketamine use; however, there are few reports of cystitis caused by intravenous esketamine in children.

**Methods:**

We report a 9-year-old boy diagnosed with NORSE, who was treated with esketamine as an anesthetic to terminate status epilepticus. He received a continuous intravenous infusion of esketamine, starting at 2 mg/kg/h and gradually increasing to a maximum of 5 mg/kg/h. The dose was reduced starting on day 9 with no apparent convulsive seizure. On day 13, he developed fluid imbalance, and an ultrasound revealed bladder wall thickening. By day 16, he exhibited gross hematuria.

**Results:**

According to the Naranjo adverse reaction probability scale, esketamine was the possible cause of the Adverse Drug Reaction (ADR). After stopping esketamine and initiating urine alkalinization with sodium bicarbonate, his urinalysis and sedimentation rate normalized.

**Discussion:**

Hemorrhagic cystitis may occur during continuous high-dose intravenous esketamine infusions. Early multidisciplinary monitoring of lower urinary tract symptoms and implementation of preventive measures in pediatric patients is essential to avoid serious urinary tract complications.

## Introduction

Ketamine, an N-methyl-D-aspartate (NMDA) receptor antagonist, has been suggested for the termination of status epilepticus as anesthetic (1). Since the first clinical report of ketamine-induced urinary tract complications in 2007, it has now recognized that long-term abuse of ketamine can lead to ketamine-associated cystitis (KAC) (2). This condition includes severe lower urinary tract symptoms such as reduced bladder capacity, dysuria, hematuria, and lower abdominal or suprapubic pain (3). Although many cases of long-term ketamine abuse causing KAC have been reported, cases of intravenous esketamine-induced cystitis remain rare. Esketamine, the S-enantiomer of ketamine, exhibits twice more the NMDA receptor affinity than racemic ketamine. It has both analgesic and anesthetic effects, causes less cognitive impairment than racemic ketamine, and is increasingly used in psychiatry and clinical anesthesia (4, 5). It displays promise in treating new-onset refractory status epilepticus (NORSE). This report represents a rare case of hemorrhagic cystitis in a child following prolonged intravenous esketamine treatment for NORSE.

## Case presentation

A 9-year-old boy (weight: 24 kg) was admitted to a local hospital with fever and seizures. The patient was treated with mannitol, cefazolin, methylprednisolone, and acyclovir for one week. However, he presented with lethargy, drowsiness, and was unresponsive and experienced four generalized tonic-clonic seizures. Due to non-significant clinical improvement, he was transferred to our pediatric intensive care unit (PICU) under sedation. On admission, he had a low-grade fever and was unconscious.

The vital signs were as follows: Temperature of 37.6° C, a heart rate of 85 beats/min, a respiratory rate of 22 breaths/min, and a blood pressure of 105/63 mmHg. Physical examination revealed an unresponsive and lethargic mental state, sluggish pupillary light reflex, pale lips, a slightly pale complexion, and neck stiffness.

Initially, the child presented with recurrent fever, drowsiness, and lethargy, accompanied by grand mal seizures. Neuroimaging studies (brain MRI and CT) performed prior to CSF analysis showed no significant abnormalities. Cerebrospinal fluid (CSF) analysis showed: a clear and colorless appearance with a weakly positive qualitative test for protein, and the nucleated cell count of 4 × 10^6^/L. Based on the clinical manifestations and the current laboratory findings, the patient is preliminarily diagnosed with viral encephalitis., The patient received intravenous immunoglobulin (IVIG) therapy, acyclovir for antiviral therapy, and mannitol to reduce intracranial pressure. On day 2, the patient experienced a generalized tonic-clonic seizure lasting 1 min and a focal impaired awareness seizure lasting over 10 min. A midazolam dose of 0.1 mg/kg/h and a remifentanil dose of 2 µg/kg/h were administered for sedation and analgesia. The patient's electrolyte levels—calcium 2.32 mmol/L, sodium 135.4 mmol/L, potassium 4.41 mmol/L, chloride 98.5 mmol/L—and glucose 5.07 mmol/L are all within normal limits. Further diagnostic evaluations included next-generation sequencing (NGS), CSF analysis, and electroencephalography (EEG). Apart from a slightly elevated CSF protein level, immunological, paraneoplastic, and infectious disease screenings produced negative results.

Despite increasing the midazolam dose to 0.4 mg/kg/h, the patient continued to experience frequent seizures occurring 3–5 times per day. On day 4, levetiracetam and oxcarbazepine were initiated as antiepileptic drugs (AEDs). However, seizures persisted and were accompanied by apnea, necessitating mechanical ventilation. On day 10, propofol was initiated at a dose of 20 µg/kg/min, resulting in seizure control but also causing bradycardia. On day 11, propofol was discontinued and replaced with esketamine at a dose of 2 mg/kg/h for seizure control. Bedside video EEG revealed ongoing epileptic discharges, severe brain injury, and predominant delta activity. The esketamine dose was subsequently increased to 3 mg/kg/h on day 12. Despite dose adjustment, the patient continued to experience frequent focal seizures, presenting as tonic jerks in the left lower limb. On day 17, therapeutic drug monitoring revealed subtherapeutic levels of oxcarbazepine metabolites (2.04 µg/ml) and levetiracetam (4.31 µg/ml), prompting an increase in AED dosage. After six days of continuous esketamine infusion at 3 mg/kg/h, the child experienced no generalized tonic-clonic seizures but continued to have recurrent focal impaired awareness seizure. Therefore, on day 18, the esketamine dose was increased to 5 mg/kg/h. By day 19, the patient experienced no significant seizure activity, allowing for a gradual reduction of the esketamine dose. Details of the primary treatment medications and their dosages are provided in Table 1.

**Table 1:** The dose of the primary drug during esketamine treatment.

Drug	Day 11	Day 12	Day 13	Day 14	Day 15	Day 16	Day 17	Day 18	Day 19	Day 20	Day 21	Day 22	Day 23	Day 24	Day 25	Day 26
Remifentanil μg/kg/h	2	2	2	2	2	2	2	2	2	2	2	2	2	2	2	2
Midazolam mg/kg/h	0.4	0.3	0.3	0.5	0.5	0.5	0.5	0.6	0.6	0.6	0.6	0.6	0.6	0.6	0.6	0.5
Esketamine mg/kg/h	2	3	3	3	3	3	4	5	4	3	3	3	3	2	2	1
Levetiracetam injection solution mg/Q12h	480	480	480	480	720	720	720	720	720	720	720	720	678	678	678	450
Ocasepine tablets mg/Q12h	240	240	240	240	300	300	300	300	300	360	360	360	450	450	450	340
Dexmedetomidineμg/kg/h															0.2	0.2

On admission, the boy weigh 24 kg; on day 23, he weigh 21.5 kg. Intravenous infusion of esketamine started at 2 mg/kg/h and gradually increasing to a maximum of 5 mg/kg/h. The dose was reduced starting on day 19 with no apparent convulsive seizure.

**Figure 1:**
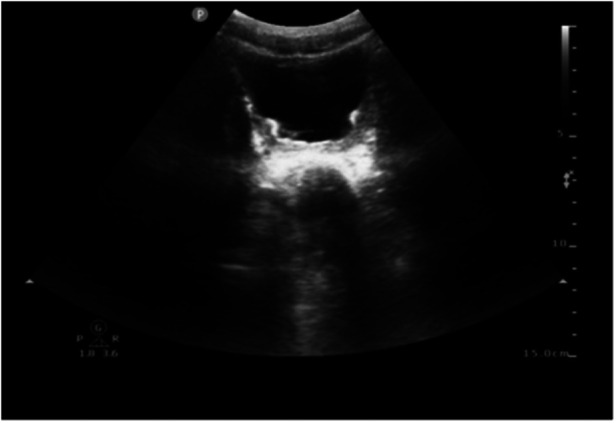
Ultrasound image of bilateral kidneys/ureters/bladder. Ultrasound revealed insufficient bladder filling and an irregular thickening of the posterior bladder wall measuring up to 10 mm.

On day 23, the fluid balance assessment revealed a mismatch between the input of 2,476 ml and the output of 1,580 ml. Despite negative fluid balance, renal function tests (RFT) showed normal serum creatinine (29 μmol/L), ruling out acute kidney injury and directing focus to localized bladder pathology. The routine blood test showed an absolute eosinophil count of 30 cells/μl, which is within the normal range. Bladder ultrasound revealed inadequate bladder filling, bladder wall thickening (up to 6 mm), and a 22 × 7 mm hyperechoic mass suggestive of a calculus. On day 25, bedside EEG indicated a reduction in epileptic discharges, suggesting a further reduction of esketamine, midazolam, levetiracetam, and oxcarbazepine doses. Urinalysis revealed pyuria and microscopic hematuria. By day 27, the patient presented with mild erythema at the urethral meatus, and small red blood clots measuring 0.2 × 0.2 cm were observed in the diaper, indicating hematuria. A repeat ultrasound revealed insufficient bladder filling and an irregular thickening of the posterior bladder wall measuring up to 10 mm, as illustrated in Figure 1. The onset of hematuria and cystitis following 16 days of continuous high-dose intravenous esketamine infusion suggested a diagnosis of hemorrhagic cystitis, possibly due to esketamine. Based on the current literature and post-marketing surveillance data, midazolam, levetiracetam, and oxcarbazepine are not associated with hemorrhagic or inflammatory cystitis. According to the Naranjo adverse drug reaction probability scale in Table 2, the cystitis was classified as possibly related to esketamine use, with a score of 4 (6). Management included urine alkalization using a sodium bicarbonate dose of 40 ml, administered once daily for 12 days. By day 40, both urinalysis and urine sediment examinations had returned to normal. A summary of the urinalysis results during treatment is provided in Table 3.

**Table 2:** Naranjo ADR probability scale —items and score.

To assess the adverse drug reaction please answer the following questionnaire and give the perinent score				
Questions	Yes	No	Do not know	Score
1. Are there previous conclusive reports on this reaction?	+1	0	0	0
2. Did the adverse event appear after the suspected drug was administered?	+2	−1	0	+2
3. Did the adverse reaction improve when the drug was discontinued or a specific antagonist was administered?	+1	0	0	+1
4. Did the adverse reaction reappear when the drug was readministered?	+2	−1	0	0
5. Are there alternative causes (other than the drug) that could on their own have caused the reaction?	−1	+2	0	0
6. Did the reaction reappear when a placebo was given?	−1	+1	0	+1
7. Was the drug detected in the blood (or other fluids) in concentrations known to be toxic?	+1	0	0	0
8. Was the reaction more severe when the dose was increased or less severe when the dose was decreased?	+1	0	0	0
9. Did the patient have a similar reaction to the same or similar drugs in any previous exposure?	+1	0	0	0
10. Was the adverse event confirmed by any objective evidence?	+1	0	0	0
Total score	4			

Naranjo scale ranges from −4 to +13 and the drug reaction was considered definite if the score was ≥9, probable if 5–8, possible if 1 to 4, and doubtful if ≤0. Causality assessment was done using Naranjo's scale and a score of 4 indicates a possible causal relationship.

**Table 3:** Urine routine and sediment examination indexes during treatment.

Urinalysis parameters	Day 23	Day 26	Day 27	Day 30	Day 31	Day 33	Day 40
Blood in urine	–	+++	+++	+++	+++	++	–
Protein in urine	–	++	+++	+++	++	+	–
Urine pH	5.0	7	6.5	8.0	7.5	7.5	7.5
Red blood cell count/ul	5.0	1,327.3	6,853	3,957.1	628.2	486.2	0
White blood cell count/ul	1.1	50.1	98.4	147.7	89.1	134.3	3.5
Bacteria/ul	146.5	1,443.8	396.2	169.4	3,488.7	38	16.6

Urinalysis revealed pyuria and microscopic hematuria on day 26. By day 40, both urinalysis and urine sediment examinations had returned to normal.

## Discussion

NORSE management remains challenging due to the lack of standardized treatment-specific guidelines. Therapy involves aggressive, multimodal options, including the use of anesthetic agents. While agents such as midazolam, propofol, and barbiturates are frequently used, their efficacy may be limited in prolonged cases (7). Ketamine and its S-enantiomer, esketamine, have emerged as potential salvage therapies for NORSE due to NMDA receptor antagonism and associated neuroprotective properties (8). A systematic review indicated that ketamine reduced seizure duration and demonstrated higher safety, though evidence in pediatric NORSE remains limited (9).

## Esketamine and hemorrhagic cystitis: causality in this case

The temporal association between esketamine infusion administered at 3 mg/kg/h for 16 days and the onset of hemorrhagic cystitis provides strong evidence supporting drug-induced bladder injury. Hematuria and bladder wall thickening appeared after prolonged high-dose administration, resolving upon dose reduction and urine alkalinization. A Naranjo adverse drug reaction probability scale score of 4 ‘possible’ further strengthens causality. Alternative causes were systematically excluded: There was no evidence of urinary tract infection (negative cultures, absence of fever), and nephrotoxic agents, such as mannitol, which did not exhibit a temporal association with symptom onset. Notably, the presence of hyperechoic deposits may represent the crystallization of esketamine metabolites, a process potentially exacerbated by acidic urine pH. The urine analysis from Day 23 (when a fluid imbalance was noted) was added, showing a urine pH of 5.0. This finding supports the hypothesis that acidic urine may precipitate esketamine.

## Mechanistic insights from ketamine-related cystitis

Although urinary toxicity is more commonly reported with racemic ketamine, the esketamine S-enantiomer shares metabolic and pharmacologic pathways with it, which suggests comparable urologic risks. Esketamine undergoes hepatic metabolism to norketamine, a metabolite known to accumulate in bladder mucosa, where it drove extensive urothelial tissue damage, resulting in chronic inflammation (10). The urothelium presents as denuded and contains eosinophils and mast cells due to inflammatory response (11). Animal studies have demonstrated that ketamine disrupts the urothelial barrier by reducing tight junction protein expression, facilitating penetration of urinary toxins into the submucosa, triggering an inflammatory response, and causing hemorrhagic cystitis (12). In this patient, sustained high-dose esketamine exposure may have exceeded renal clearance capacity, exacerbating direct mucosal toxicity. Additionally, signs of dehydration reflected by an imbalance in fluid intake and output result in urine having concentrated metabolites, accelerating crystalluria and hematuria.

## Dose-duration threshold, gender, and pediatric vulnerability

Esketamine-induced bladder toxicity appears to be both dose- and duration-dependent (13). In adult refractory depression trials, urinary symptoms such as dysuria and hematuria were reported in 1%–3% receiving long-term intranasal esketamine (14); however, pediatric data on intravenous administration remains sparse. A systematic review of ketamine safety in status epilepticus reported an average infusion rate of 2–4 mg/kg/h, yet none of the included studies documented urinary tract toxicity (9). The present case highlights a potential toxicity threshold, with esketamine administered at 3 mg/kg/h for 16 days—far exceeding standard protocols for NORSE management where theoretically typical dosing ranges from 1 to 2 mg/kg/h. Analysis of FDA Adverse Event Reporting System (FAERS) data indicates that ketamine-associated toxicities, including bradycardia, cystitis, and agitation, were more frequent in males, indicating sex-based differences in adverse event profiles (15).

Compared to adults, children may be more vulnerable to ketamine-induced bladder injury due to anatomical and developmental differences. The pediatric bladder urothelium is thinner and structurally immature, with underdeveloped umbrella cell junctions and a less established glycosaminoglycan barrier, potentially allowing deeper penetration of ketamine metabolites (13). Moreover, the higher proliferative activity of basal cells and greater microvascular fragility in children may contribute to more pronounced inflammatory and hemorrhagic responses. Developmental pharmacokinetic factors, such as reduced CYP3A4-mediated metabolism, might also enhance local toxin exposure.

In this case, despite subtherapeutic levels of adjunctive AEDs (levetiracetam, oxcarbazepine), esketamine's local bladder toxicity likely predominated.

## Clinical implications for esketamine (and ketamine) in NORSE management

1. **Proactive Monitoring**: In patients receiving prolonged esketamine infusions for more than 7 days, especially exceeding a dose of more than 2 mg/kg/h, routine urinalysis and a bladder ultrasound scan are recommended. The detection of asymptomatic crystalluria or bladder wall thickening should prompt timely clinical intervention.
2. **Preventive Measures**: To reduce the risk of crystal formation, aggressive fluid administration volume greater than 1.5 times maintenance requirements and urine alkalinization aiming for a pH between 7.5 and 8.0 are advisable.
3. **Therapeutic Alternatives**: If discontinuation of esketamine is unfeasible, adjunctive uroepithelium protective therapies such as intravesical hyaluronic acid should be considered (12). Upon seizure control, transitioning to alternatives with less urotoxicity, such as topiramate or a ketogenic diet, should be explored (7).

## Contrasting esketamine and racemic ketamine toxicity

Although racemic ketamine was linked to a 20%–30% incidence of cystitis among chronic users, esketamine was thought to pose a lower risk due to its higher potency and reduced psychotropic side effects (4, 16). This case, however, highlights that even high-dose intravenous esketamine administered for therapeutic purposes can result in significant bladder damage. Pediatric patients may be particularly vulnerable, warranting individualized protocols.

This case has several limitations. First, due to the initial clinical focus on viral encephalitis, we did not perform serum IgE testing at the time of onset, which may have delayed recognition of an IgE-mediated hypersensitivity reaction.

## Conclusion

This case represents the first reported instance of hemorrhagic cystitis associated with prolonged high-dose esketamine infusion in a pediatric NORSE patient. Clinicians must carefully weigh the anticonvulsant advantages of esketamine against its potential for cumulative urological toxicity, especially with prolonged infusion durations. Coordinated multidisciplinary care involving neurology, nephrology, and pharmacy teams, along with early implementation of preventive measures, is crucial to addressing this often-overlooked complication.

## Data Availability

The original contributions presented in the study are included in the article/Supplementary Material, further inquiries can be directed to the corresponding author.
